# Evaluation of the NBS Unit of Resistance Based on a Computable Capacitor

**DOI:** 10.6028/jres.065A.018

**Published:** 1961-06-01

**Authors:** Robert D. Cutkosky

## Abstract

An evaluation of the unit of resistance maintained at the National Bureau of Standards, based on the prototype standards of length and time, is described. The evaluation is based on a nominally one-picofarad capacitor whose value may be calculated from its mechanical dimensions to high accuracy. This capacitor is used to calibrate an 0.01-microfarad capacitor. A frequency-dependent bridge involving this capacitor establishes the value of a 10^4^-ohm resistor. Comparison of that resistor with the bank of one-ohm resistors maintaining the NBS unit of resistance establishes that this unit is

$$\Omega_{EU}=1.000002_{3}\;\mathrm{ohms}\pm 2.1\mathrm{ppm}.$$

The indicated uncertainty is an estimated 50 percent error of the reported value based on the statistical uncertainty of the measurements and allowing for known sources of possible systematic errors other than in the speed of light, assuming that the speed of light c=2.997925×10^10^cm/sec.

## 1. Introduction

The United States national standard of resistance is maintained at the National Bureau of Standards by a group of 1-ohm manganin resistors. Values of resistance are assigned to the 1-ohm reference standards by techniques based ultimately upon the national prototype standards of length and time.

The most accurate methods which have been used for this purpose in the past made use of either self-inductors or mutual inductors whose inductances were computable from their mechanical dimensions. In principle, it is then straightforward to compare the impedance of the computable inductor at a known frequency with that of the 1-ohm reference standard resistors. In practice, this comparison is likely to involve several steps, and may not even make use of sinusoidal currents through the inductor.

An alternative method for assigning values to the reference resistors involves constructing a capacitor whose capacitance may be calculated from its mechanical dimensions. By comparing the impedance of the capacitor at a known frequency with that of the reference standard resistors one may establish the values of the reference resistors in terms of the prototype standards.

Within recent years the development of an improved computable capacitor [1, 2, 3, 4]^1^ and improved methods for the precise comparison of capacitances [5, 6] have made the latter process appear to be the more fruitful.

The method involves stepping up from a 1-pf three-terminal computable capacitor to two 0.01-*μ*f three-terminal capacitors. The step-up is made in four steps, using a transformer whose nominally 10:1 ratio may be accurately measured. The average admittance of the two 0.01-*μ*f capacitors at an angular frequency of 10^4^ radians per second is compared with the average admittance of two 10^4^-ohm shielded a-c resistors using a bridge network to be described. These measurements serve to assign values to the 10^4^-ohm a-c resistors in terms of the prototype standards.

A conventional d-c step-down from these 10^4^-ohm a-c resistors to the 1-ohm NBS reference resistors provides an absolute calibration of the 1-ohm resistors. A small correction must be applied to the results so obtained because of the a-c–d-c differences of the 10^4^-ohm resistors. This correction is determined by comparison with a special transfer resistor.

## 2. Computable Cross Capacitor

Computable capacitors of the traditional guard-ring or guard-cylinder design require, in general, the measurement of several dimensions. For example, with the guard-ring type, area, plate separation, flatness of electrodes, parallelism, and proper location of the guarded island are of major concern. Most of these dimensions have to be measured to a higher accuracy than the required overall accuracy of the computed capacitance.

The development by A. M. Thompson and D. G. Lampard of a device called by them a computable cross capacitor [1] has made possible the construction of a capacitor whose value may be computed from dimensional measurements with an accuracy considerably higher than that attainable with any previous design.

**Figure 1:**
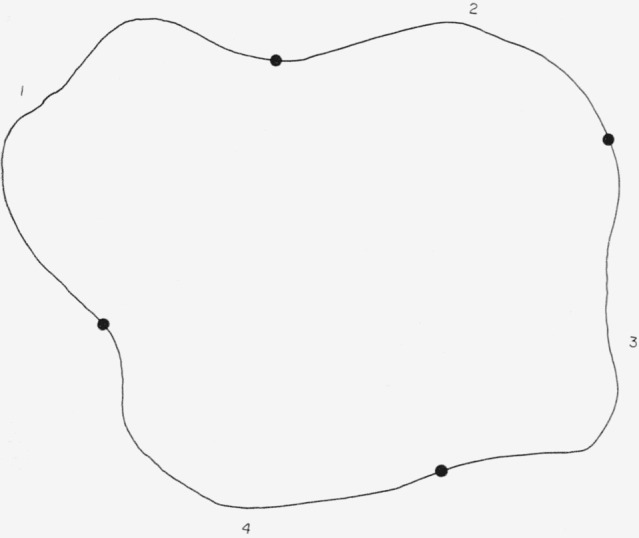
Cross section of a general cross capacitor.

A cross capacitor consists of a cylindrical arrangement of four electrodes, with cross section as shown in figure 1. Electrodes 1, 2, 3, and 4 are separated from each other by very small insulating gaps. Let *C*_1_*/L* be the direct capacitance per unit length (perpendicular to the plane of the drawing) between electrodes 1 and 3 with electrodes 2 and 4 grounded, and let *C*_2_/*L* be the direct capacitance per unit length between electrodes 2 and 4 with electrodes 1 and 3 grounded. It may be shown that in *cgs*, esu,

$$\begin{array}{c} e^{-4\pi^{2}C_{1}/L}+e^{-4\pi^{2}C_{2}/L}=1,\;\text{or in MKS units},\\ \bar{C}=\frac{1}{2}(C_{1}+C_{2})=\epsilon_{0}L(\ln2/\pi )[1+0.087{\left(\Delta C/\bar{C}\right)}^{2}]\\ +O\{{\left(\Delta C/\bar{C}\right)}^{4}\}]\;\text{farads} \end{array}$$ (1)

where ***ϵ****_0_*=1*/μ_0_c*^2^,* c* is the speed of light, and Δ*C= C*_2_−*C*_1_. The notation “ O” means that all remaining terms in eq (1) are fourth order or higher in $\Delta C/\bar{C}$, and their sum approaches zero as $K{\left(\Delta C/\bar{C}\right)}^{4}$ as $\Delta C/\bar{C}$ tends to zero. This relationship allows $\bar{C}$ to be calaculated from a single measured length *L.* One finds that if *C*_1_ *≈ C*_2_, then $\bar{C}/L\approx 2$ pf/meter ≈ 0.05 pf/in.

**Figure 2:**
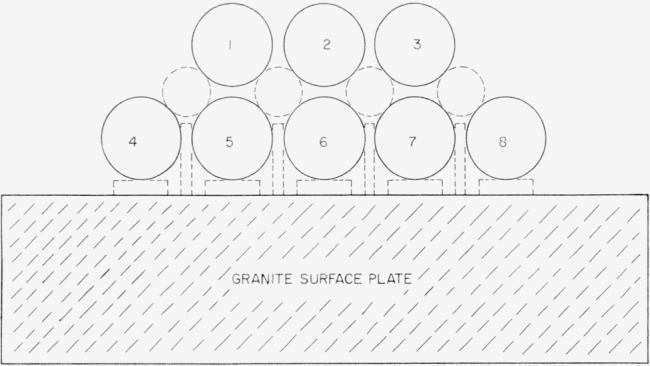
Cross section of the NBS cross capacitor.

**Figure 3:**
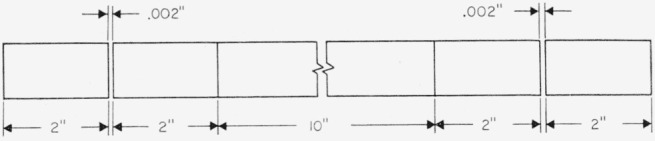
Side view of the NBS cross capacitor detector electrodes.

The requirements that both $\bar{C}$ and *L* be easily measurable have led to the use at NBS of two cross capacitors in parallel, each about 10 in. long, to produce a capacitance of about 1pf [6]. Figure 2 shows the cross section of the NBS 1-pf computable cross capacitor, which is housed in an evacuated chamber. Electrodes 1, 2, 5, and 6; and electrodes 2, 3, 6, and 7 of the two cross capacitors are steel bars ⅞ in. in diameter supported with accurately ground glass slips and disks, shown dotted in the figure, resting on a precision granite surface plate. The bottom row of bars is held together with a phosphor-bronze coil spring between bars 4 and 8 at each end of the assembly. The glass spacers are all located within guard sections at the ends of the capacitor, so that no solid dielectric appears between the bars within the active length of the capacitor. Figure 3 shows a side view of electrode 6, which is similar to electrode 2. The assembly is constructed of precision gage blocks having circular cross sections, with wrung joints between the 10-in. bar and its adjacent 2-in. bars, and insulated with firmly cemented 0.002-in. mica spacers between each pair of 2-in. bars. The outer 2-in. bars constitute the guard sections, and are grounded. The composite 14-in. central section defines the length of the cross capacitor, and extends roughly between the centers of each insulated gap. Electrodes 1, 3, 5, and 7 are approximately 18 in. long, and so extend well beyond the active sections of bars 2 and 6.

Additional shields not shown in figure 2 eliminate capacitive coupling between electrodes around the outside of the grounded electrodes. This is essential, since (1) deals only with the capacitance associated with the region in the interior of the electrode system.

One capacitance measurement is made, with electrodes 1, 3, and 6 grounded, of the direct capacitance between electrodes 5 and 7 in parallel and the central section of electrode 2. Another measurement is made, with electrodes 5, 7, and 2 grounded, of the capacitance between electrodes 1 and 3 in parallel and the central section of electrode 6. If we neglect, for the moment, the small second order correction $0.087{\left(\Delta C/\bar{C}\right)}^{2}$ in eq 1, the average of these two capacitances is equal to 2×*ϵ_0_L*′ln2/′*π*; where *L*′ is the average length of the two central sections of electrodes 2 and 6, or approximately 14 in. If the 10-in. gage blocks are removed from electrodes 2 and 6 and the 2-in. bars on either end are wrung directly together, a new cross capacitor is formed with a length differing from the first by twice the average length of the 10-in. bars, or about 20 in. The difference between the two cross capacitances so measured is not affected by irregularities or lack of perfect centering of the bars near the insulated gap, provided that the end sections are not rotated or otherwise disturbed when the 10-in. bars are removed or replaced.

The small discontinuities caused by the slightly-rounded ends of the gage blocks produce only second-order errors in the capacitance computed from eq (1). These errors have been investigated experimentally by introducing additional discontinuities on the bars.

Between May 12 and May 18, 1960, and again between July 21 and July 27, 1960, two series of measurements with the NBS computable cross capacitors were made to establish the value of a fixed, 1-pf, air-dielectric, three-terminal, reference capacitor; NBS 89790-B. This capacitor was maintained in an oil bath at a constant temperature near 25 °C.

**Figure 4:**
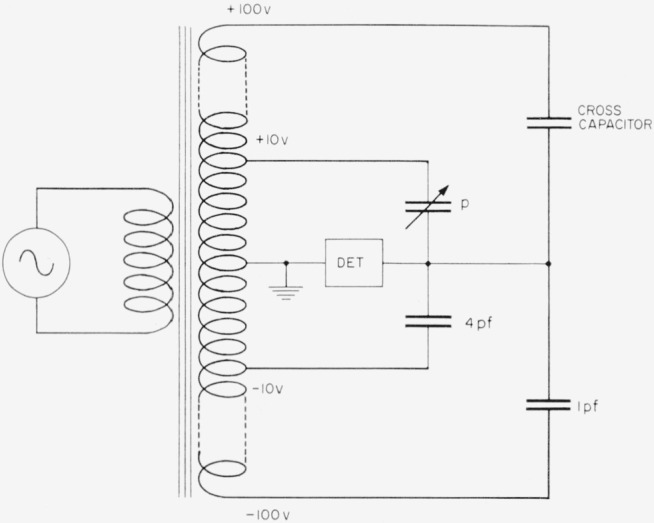
Inductively coupled ratio arm bridge used with the NBS cross capacitor.

A transformer ratio-arm bridge was used for this purpose with the circuit shown in figure 4. The cross capacitor with the 10-in. bars in place has a capacitance slightly under 1.4 pf so that the bridge may be balanced with a small adjustable capacitance *p* as shown, and a small adjustable conductance not shown, which may be placed on either side of the bridge. The bridge components have been described in detail elsewhere [6] and will not be elaborated upon here.

The bridge circuit for the measurement with the 10-in. bars removed is similar to figure 4 except that the cross capacitance is now about 0.4 pf, so the 1-pf capacitor in the lower portion of the circuit must be removed. The 4-pf capacitor consists of 4 one-pf capacitors which may each be compared directly with the 1-pf standard, and then connected in parallel. Since the adjustable capacitor has a relatively high reading, it is also necessary to calibrate it for each measurement.

All of the above steps are straightforward and can be done in a symmetrical fashion (calibration, cross-capacitor measurement, and recalibration) in less than 30 min by an experienced operator.

Before and after the determination, the 1-pf capacitor used is compared with the 1-pf reference capacitor in the oil bath.

**Figure 5:**
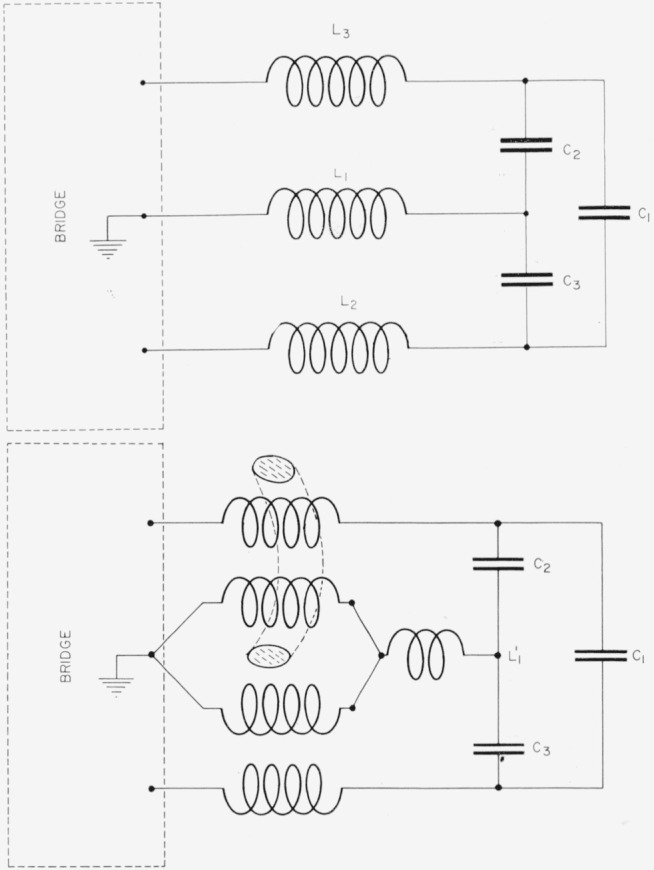
Three-terminal capacitor *C*_1_ connected to a bridge with three leads, with four leads and using a high permeability core (See text).

Although small direct (three-terminal) capacitances are much easier to measure than small two-terminal capacitances because of the virtual elimination of connection problems, a particularly troublesome source of error arises when capacitance to ground is very high, as is the case with the NBS cross capacitor. Figure 5 shows a three-terminal capacitor with direct capacitance *C*_1_ connected to a bridge with leads of inductance *L*_1_ *L*_2_, and *L*_3_. It may be shown that the effective capacitance seen by the bridge is given by

$$C_{eff}\approx C_{1}\left[1-\frac{\omega^{2}\left(C_{2}C_{3}\right)}{C_{1}}L_{1}+\omega^{2}C_{3}L_{2}+\omega^{2}C_{2}L_{3}+\omega^{2}C_{1}\left(L_{2}+L_{3}\right)\right].$$

For *C*_1_, *C*_2_, and *C*_3_ all less than 1000 pf, the important error, expressed as an additive term, is −*ω*^2^*L*_1_*C*_2_*C*_3_; which is independent of *C*_1_ and hence relatively more important for a very small *C*_1_. Thompson [5] has shown that if the capacitor is treated as a four-terminal network as in figure 5 with a high permeability core linking one ground lead with one active electrode lead, the troublesome error term is eliminated. This technique has been used throughout the measurements reported here. It is impossible with the NBS cross capacitor to completely eliminate all common ground lead inductance in this way, and a small inductance *L*_1_*′* remains in the cross capacitor circuit. We have found it necessary to measure *C_eff_* as a function of frequency and extrapolate to zero frequency under the assumption that the error is proportional to *ω*^2^. With most of *L*_1_ eliminated by the use of high permeability cores, the factor *ω*^2^*L*_1_′*C*_2_*C*_3_ is very small, and the d-c value may be determined to high accuracy.

Table 1 shows the results of two complete series of measurements on the NBS computable cross capacitor, based on the assumption that the reference capacitor in the oil bath was exactly 1 pf.

The correction term in the measured capacitance $0.087{\left(\Delta C/\bar{C}\right)}^{2}$ is less than two parts in 10^7^ and has been neglected. The calculated cross capacitance also appears in table 1, based on the measured lengths of the 10-in. gage blocks, and taking the speed of light c=2.997925×10^10^ cm/sec as adopted recently by the IGGU and the URSI. Combining these figures we obtain true values for the reference capacitor at the time of measurement. The difference between the May and July figures reflects a shift in the temperature of the oil bath which occurred some time in June, and has no effect on the final results quoted in this paper. The stability of the reference capacitor and the closeness of temperature regulation during each of the determinations was sufficient to reduce any error arising from the variability of the reference capacitor considerably below 1 ppm.

## 3. Capacitance Step-Up

**Figure 6:**
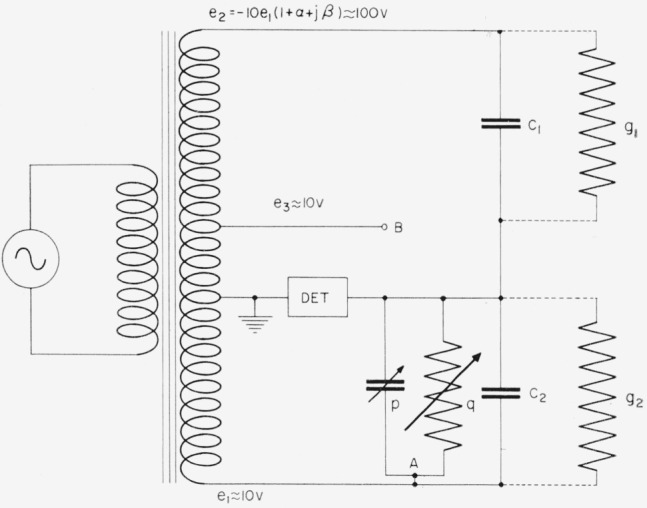
Ten-to-one inductively-coupled ratio arm bridge for stepping up from 1 pf to 0.01 μf in four steps.

The bridge for comparing capacitive reactance with resistance, to be described in a later section, requires the use of two 0.01-*μ*f capacitors. These capacitors must be calibrated in terms of the 1-pf reference capacitor. A transformer ratio-arm bridge with a nominally 10:1 ratio is used for this calibration in the circuit of figure 6.

The balance conditions are, to a sufficiently close approximation,

$$C_{2}=10\;C_{1}\left(1+\alpha \right)-p,\;\text{and}\;g_{2}=10\;g_{1}-10\omega C_{1}\beta -q,$$

where *p* and *q* are small adjustable admittances for balancing the bridge. One may connect *p* and *q* to point “*B*” instead of “*A*” to change their apparent signs if this is needed to obtain a balance.

Shielding completely surrounds each of the capacitors in the circuit in such a way that the measurement compares the direct capacitances *C*_1_ and *C*_2_. This technique allows the direct capacitance of the 10-pf capacitor to be determined relative to the 1-pf reference capacitor. The 10-pf capacitor is in turn compared with a 100-pf capacitor, to reach after four such steps the 0.01-*μ*f mica capacitors used in the resistance bridge. All of the step-up capacitors are located in a regulated oil bath. The 0.01-*μ*f capacitors as well as the 1000-pf capacitor are sufficiently large to require correction for the equivalent series inductances of the transformer. The total inductance correction was found to be two parts in 10^7^ for each 0.01-*μ*f capacitor [6].

It was desired that the total uncertainty in the step-up be less than one part in 10^7^, which requires that the transformer ratio 10(1 + *α+jβ*) be known to an accuracy of about two parts in 10^8^. A procedure for calibrating such a transformer to the required accuracy has been described [7] which involves the use of 11 nominally equal three-terminal capacitors having their detector electrodes connected together. Provision is made for switching any one of the capacitors to the high voltage side of the transformer while the other 10 are in parallel on the low voltage side. The bridge was balanced with a small admittance on one side or the other of the bridge with each of the 11 capacitors in turn on the high voltage transformer winding. After applying small corrections for lead impedances one obtains from this series of measurements values for *α* and *β* in the expression for the transformer ratio.

## 4. Resistance-Capacitance Bridge

Several bridges capable of being used for the comparison of resistance with capacitive reactance have been used in the past, one of the most popular being the Wien bridge. The Wien bridge, like many frequency dependent bridges, is difficult to shield against stray pickup. The difficulty comes from the fact that one arm of the bridge contains a resistor and a capacitor in series. If this arm is shielded, leakage currents from their common point to the shield produce errors which are difficult to evaluate with high accuracy.

### 4.1. Bridge Equations

**Figure 7:**
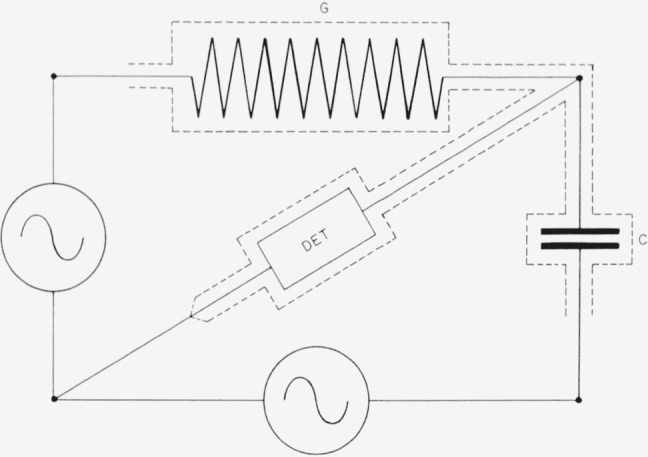
Basic bridge for comparing conductance with capacitive susceptance.

In theory it is simple to compare resistance with capacitive reactance provided that one may obtain two a-c voltage sources exactly 90° out of phase and exactly equal in magnitude. Figure 7 shows two such sources used to compare a capacitance *C* with a conductance *G.* The balance condition is *G=ωC* The components may be readily shielded as shown with dotted lines; leakage paths to ground are either across the detector, which at balance has no voltage on it; or across one of the generators, which is assumed to have a very low impedance. Although it has not been possible to construct voltage sources with the required phase and magnitude relationships, accurate low impedance voltage sources equal in magnitude and 180° apart in phase are readily procurable from a center-tapped transformer.

**Figure 8:**
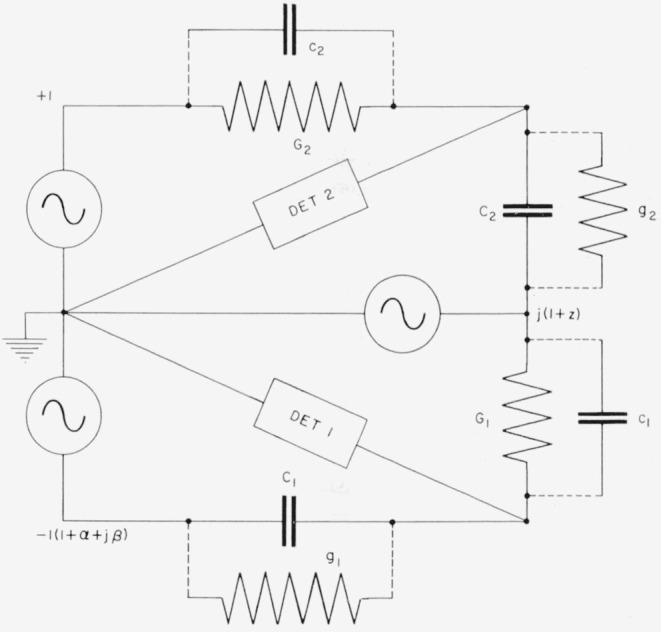
Double bridge for comparing conductance with capacitive susceptance.

A double bridge using the principles of figure 7 may be constructed with the circuit of figure 8, which shows all voltages referred to one generator as reference.^2^ The voltages +1 and *−*1 (1+*α+jβ*) are approximately equal and 180° out of phase, with *α* and *β* representing magnitude and phase angle errors respectively. The voltage *j*(1 + *z*) is approximately 90° out of phase with each of the other generators. The small complex number *z* represents the departure of the generator from its nominal value.

Figure 8 also shows the residual leakage conductances *g*_1_ and *g*_2_ of the main capacitors *C*_1_ and *C*_2_, and the stray capacitances *c*_1_ and *c*_2_ of the main conductance standards *G*_1_ and *G*_2_.

This circuit is used with *C*_1_ ≈ *C*_2_ ≈ 0.01 *μ*f *G*_1_ ≈ *G*_2_ ≈ 10^−4^ mhos, and *ω* ≈ 10^4^ radians per second (about 1592 c/s).

With both bridges balanced we have the conditions

$$j\omega c_{2}+G_{2}=-j\left(1+z\right)\left(j\omega C_{2}+g_{2}\right)$$

and

$$\left(1+\alpha +j\beta \right)\left(j\omega C_{1}+g_{1}\right)=j\left(1+z\right)\left(j\omega c_{1}+G_{1}\right).$$

Eliminating *z* from these equations we have

$$\left(j\omega c_{2}+G_{2}\right)\left(j\omega c_{1}+G_{1}\right)=-\left(1+\alpha +j\beta \right)\left(j\omega C_{2}+g_{2}\right)\left(j\omega C_{1}+g_{1}\right).$$

Separating real and imaginary parts we find

$$G_{1}G_{2}=\left(1+\alpha \right)\omega^{2}C_{1}C_{2}+\beta \left(\omega C_{1}g_{2}+\omega C_{2}g_{1}\right)-\left(1+\alpha \right)g_{1}g_{2}+\omega^{2}c_{1}c_{2},$$

and

$$\omega \left[c_{1}G_{2}+c_{2}G_{1}\right]+\left(1+\alpha \right)\omega \left[C_{1}g_{2}+C_{2}g_{1}\right]=\beta \omega^{2}C_{1}C_{2}-\beta g_{1}g_{2}.$$

It may be shown that if all residual parameters are small, we may write approximately

$$\bar{G}=\left(1+\frac{1}{2}\alpha \right)\omega \bar{C}$$ (2)

and

$$\omega \bar{c}\bar{G}+\omega \bar{C}\bar{g}=\frac{1}{2}\beta \omega^{2}{\bar{C}}^{2}$$ (3)

where bars over letters signify the average of the two values involved.

**Figure 9:**
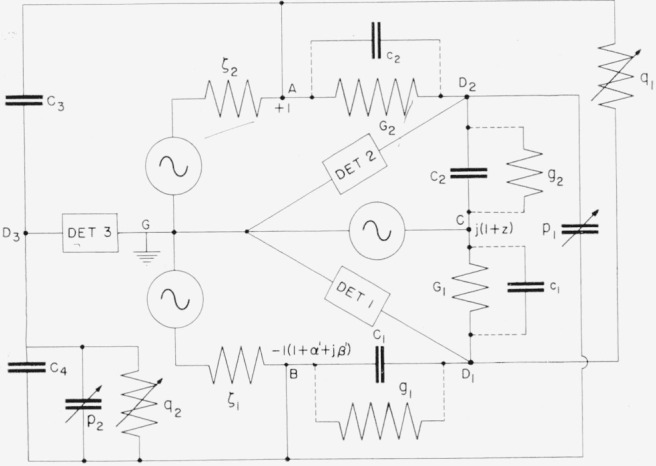
Complete bridge with auxiliary components.

The voltage appearing at the terminals of detector 1 may be reduced to zero by making either *C*_1_ or *G*_1_ and either *c*_1_ or *g*_1_ adjustable, and similarly for detector 2. One finds that even if *z* is zero, either *c*_1_ or *g*_1_ and either *c*_2_ or *g*_2_ must be negative. As shown in figure 9, this is accomplished by connecting an adjustable conductance *q*_1_ from point *A* to *D*_1_ and an adjustable capacitance *p*_1_ from point *B* to *D*_2_. The main capacitors *C*_1_ and *C*_2_ consist of the 0.01-*μ*f mica capacitors in parallel with adjustable capacitors having ranges of 1 pf. With these provisions eq (3) is replaced by

$$\omega \bar{c}\bar{G}+\omega \bar{C}\bar{g}-\frac{1}{2}\left(\omega p_{1}\bar{G}+\omega \bar{c}q_{1}\right)=\frac{1}{2}\beta \omega^{2}{\bar{C}}^{2}.$$ (4)

It is apparent from figure 9 and the above equations that *C*_2_ is the effective capacitance including the correction resulting from self inductance between junctions *C* and *D*_2_, and *G*_1_ must include the series resistance correction between junctions C and *D*_1_. If the open circuit transformer-ratio parameters *α* and *β* are used, *C*_1_ and *G*_2_ must include the effects of impedances *ζ*_1_ and *ζ*_2_, measured from junctions *D*_1_ and *D*_2_ respectively, through the transformer to the ground point *G.* To avoid the problems of accurately measuring the transformer impedances, we have found it profitable to measure the loaded rather than the open circuit transformer ratio between *A* and *B* to *G* while the bridge is balanced. Using the values so obtained for *α′* and *β*′, the required values of *G*_2_ and *C*_1_ include impedances only to junctions *A* and *B.*

The loaded transformer ratio is easily measured with a third bridge involving *C*_3_, *C*_4_, and detector 3 on the left of figure 9. This bridge is balanced with the circuit as shown and rebalanced after interchanging *C*_3_ and *C*_4_. The readings of *p*_2_ and *q*_2_ are called *p*_2_′and *q*_2_′ before interchanging, and *p*_2_*″* and *q*_2_*″* after interchanging. The transformer parameters *α′* and *β′* are calculated from the formulas

$$\alpha \prime =-\frac{p_{2}^{\prime }+p_{2}^{\prime \prime }}{C_{3}+C_{4}}\;\text{and}\;\beta \prime =\frac{q_{2}^{\prime }+g_{2}^{\prime \prime }}{\omega \left(C_{3}+C_{4}\right)}.$$

The signs of *p*_2_ and *q*_2_ are positive if connected to *B* as shown, and negative if connected to *A.* It is found that for this bridge, with the loading shown in figure 9, *α*′ ≈ + 17×10^−6^ and *β*′ ≈ −16×10^−6^.

### 4.2. Physical Bridge Arrangement

**Figure 10:**
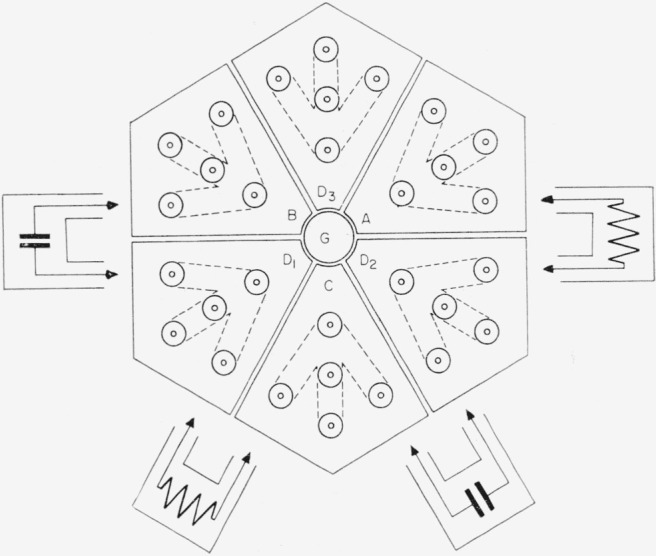
Diagram of bridge center.

In order to minimize current loops and localize the junction points *A, D*_2_,* C, D*_1_ *B*, and *D*_3_, a hexagonal bridge center was constructed of copper as shown in figure 10. Each segment consists of five coaxial connectors with their shields bolted to a ¼-in. copper top plate and their inner conductors connected by means of a copper plate. These six connecting plates shown dotted in the figure, are shielded from each other so that capacitance and conductance between segments arises only from external elements that are connected between pairs of coaxial connectors in the various segments. The intersections of the arms of the copper connecting plates define the junction points referred to in the discussion of figure 9. The bridge center is located just above the liquid level in the middle of a large temperature-regulated oil bath.

The voltage sources are connected to the bridge center points *A, B*, and *C* by heavy wires passing through a tube that is welded to the bottom of the oil bath tank and extends above the liquid level beneath the center. The 10^4^-ohm resistors and the 0.01-*μ*f capacitors, hermetically sealed and immersed in the oil to maintain them at a constant temperature, are connected to the center with rigid coaxial leads positioned as in figure 10.

**Figure 11:**
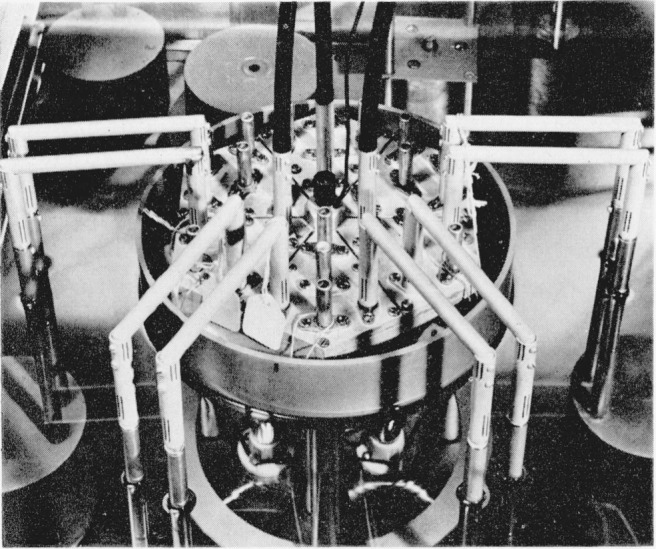
Photograph of components shown in figure 10.

**Figure 12:**
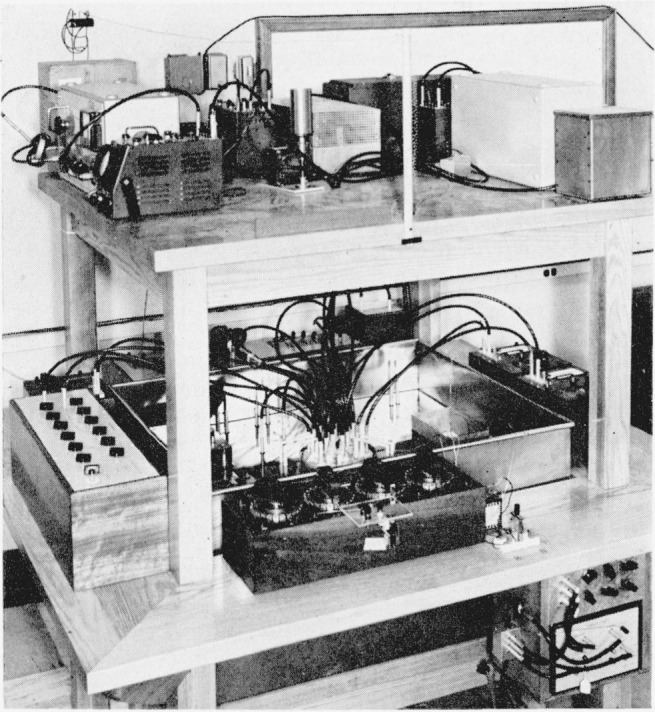
Photograph of bridge assembly for comparing conductance with capacitive susceptance.

Coaxial detector leads run from the connectors at *D*_1_, *D*_2_ and *D*_3_, upwards through an eye-level shelf to filters, amplifiers, and visual detectors. Figure 11 shows the principal features of the bridge arrangement, and figure 12 shows the auxiliary admittances in place for balancing the bridge.

Although the two voltage sources derived from a transformer are easily procurable, the third source 90° out of phase with them presented some design problems. Our equipment uses a passive phase-shifting network driven by a separate winding on the main transformer to produce this voltage. The phase-shifting network is adjustable to allow the complex components of *z* to be varied in small increments and set within a few parts in a million of zero.

**Figure 13:**
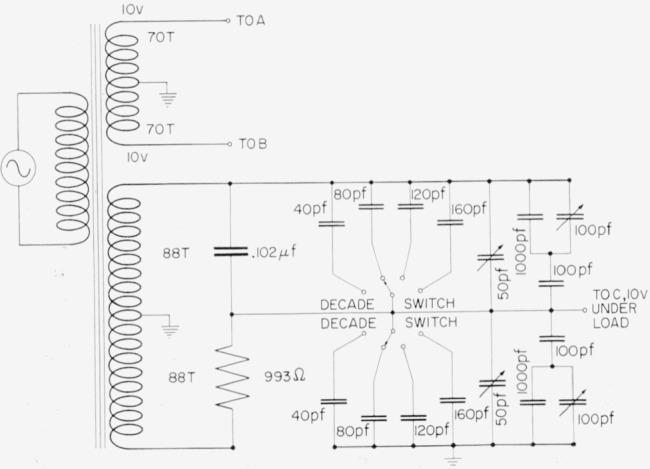
Bridge voltage sources.

The assumptions leading to the bridge equations require only that the proper voltage at junction *C* be produced and maintained, and place no restriction on the equivalent series impedance of the 90° source. However, if its impedance is too large, variation of one balancing component such as *C*_1_ in figure 9 changes not only the voltage at *D*_1_, but also the voltage at junction *C* and hence at *D*_2_. This mutual dependence between the two bridges makes it very difficult to balance both bridges simultaneously unless the impedance of the 90° source is less than 1/10 the impedance of the bridge arms, or in this case less than 1000 ohms. Based on these considerations, the circuit of figure 13 was chosen to provide the three sources for the bridge. With *A, B*, and *C* connected to the bridge the voltage at *C* may be adjusted, with the controls provided, to the proper value.

**Figure 14:**
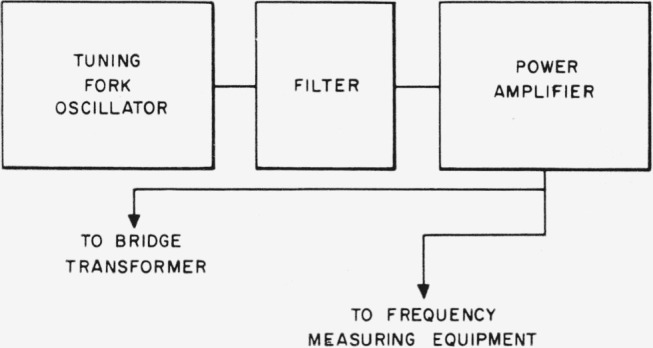
Bridge power supply.

Power for driving the bridge is provided by a commercial tuning fork oscillator driving a power amplifier as shown in figure 14. The filter is designed to reduce harmonic content of the signal. The tuning fork frequency is adjustable in a small range around *ω*=10^4^.

### 4.3. Frequency Measurement

**Figure 15:**
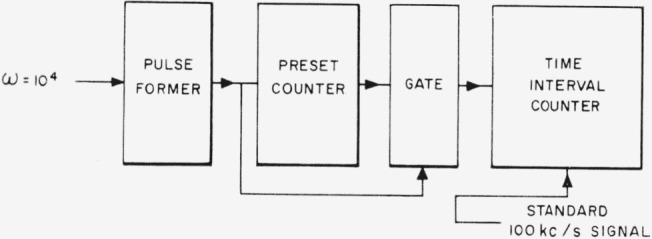
Frequency measuring system.

The frequency is measured with the circuit of figure 15. The pulse former preceding the preset counter proper is part of the preset counter assembly. The sharp pulse produced therein goes through the gate when the preset counter registers the desired number of counts, and the time interval counter measures the time between two such pulses. The preset counter is automatically reset after 10^4^ cycles at the input. For *ω*=10^4^ the time interval between pulses reaching the time interval counter is 2*π* sec, during which time a 10-Mc/s signal derived from the 100-kc/s standard frequency source is counted and displayed by the time interval counter. The system may be checked for internal consistency by using it to measure 10^4^ periods of a standard 1000-cycle signal. This equipment monitors the tuning fork output while the bridge is in use, and allows the frequency to be measured with an accuracy better than 5 parts in 10^8^.

### 4.4. Detectors

Since the bridge balance is strongly frequency dependent, the harmonics of the driving frequency may have relatively high signal levels at the detector inputs. Sharp tuning of the detectors is not sufficient to eliminate trouble from this source, since two or more harmonics may mix in the nonlinear first stages of the amplifiers to produce apparent fundamental frequency signals [8].

**Figure 16:**
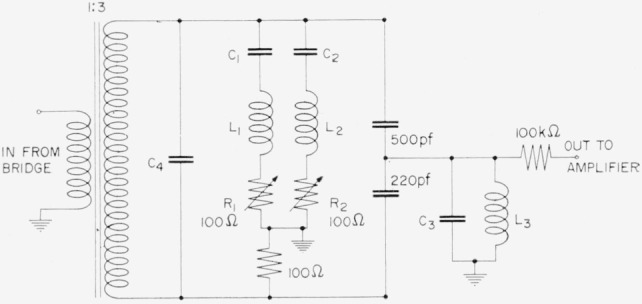
Filters for harmonic rejection.

Figure 16 shows the circuit of filters which precede the electronic amplifiers used with detectors *D*_1_ and *D*_2_. With the exception of the input transformer, all inductors are 0.1-henry commercial dust-core toroidal inductors. The input transformer must have a very high *Q* to preserve high signal strength, and was constructed from a similar inductor by removing a few turns and adding a primary; the turns ratio being chosen to give a good impedance match between the bridge and the filter. The filter forms a bridge which is balanced at the second harmonic by adjusting *C*_1_ so that *C*_1_ and *L*_1_ resonate, and by adjusting *R*_1_. Also, *C*_2_ and *R*_2_ are adjusted to produce balance at the third harmonic. Tuning the input transformer with *C*_4_ and the output circuit with *C*_3_ to the fundamental completes the adjustment of the doubly-tuned filter which attenuates harmonics higher than the third sufficiently for our purposes. The filters are housed in boxes constructed of 1/16 in. sheet steel for magnetic shielding.

It has been found that with these filters, second and third harmonic signal levels are attenuated 140 db with respect to the fundamental, and all higher harmonics by at least 95 db. Experimentation with filter orientation shows that stray pickup to the filters cannot produce an error as large as one part in 10^7^.

The effectiveness of the filters has been investigated by greatly increasing the harmonic content of the power signals reaching the bridge. For this purpose a diode rectifier was placed between the tuning-fork filter and the power amplifier. Harmonic levels at the bridge center were measured with and without this diode. It was found that although the levels of all measured harmonics were greatly increased, the bridge balance change was less than one part in 10^7^.

Following the detector filters, commercial tuned amplifiers are used, which drive the vertical deflection plates of cathode-ray tubes. The horizontal plates of the cathode-ray tubes are driven by constant amplitude sinusoidal voltages derived from the tuning-fork oscillator but passing through variable phase shifters. At balance a straight horizontal line appears on the cathode-ray tube, and the phase shifter may be adjusted so that an unbalance in a main component (*C*_1_ or *C*_2_) opens the line into an ellipse, and an unbalance in phase (*q*_1_ or *p*_2_) tilts the line. This provides a phase sensitive detection scheme which allows the bridge to be balanced with a minimum of time consumed. The detector sensitivity is sufficient to observe unbalances of one part in 10^7^ in magnitude or 10^−7^ radians in phase angle.

### 4.5. Resistance Comparisons

The bridge system described allows the mean of two a-c conductances *G*_1_ and *G*_2_ to be measured in terms of a measured frequency and two capacitors *C*_1_ and *C*_2_ whose values are obtained from a computable capacitor. The conductance standards *G*_1_ and *G*_2_ each consists of a commercial 10^4^-ohm woven-wire resistor mounted in a shielded hermetically-sealed box and placed in the oil bath. The reciprocal of their measured conductance values is a resistance, which must be stepped down and compared with the bank of 1-ohm reference resistors which maintain the unit of resistance [9].

The conductance standards *G*_1_ and *G*_2_ are completely shielded, and are measured in the a-c bridge as direct conductances. In section 5 we will describe measurements performed to evaluate the difference between their conductances as measured with 1592 c/s alternating current, and their conductances as measured with direct current. This difference may be expected to be small, and will certainly remain constant over long periods of time. We will, at this time, tentatively assume that the difference is zero, and apply a correction later.

**Figure 17:**
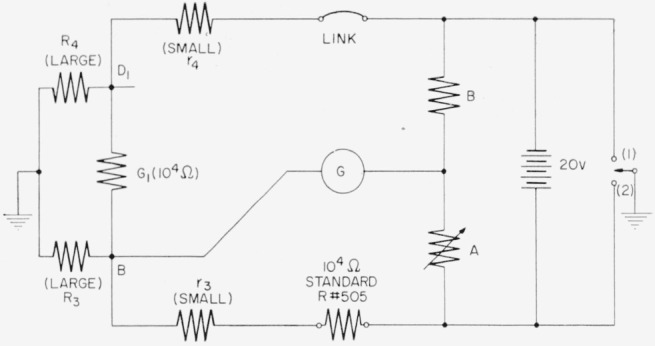
Circuit for three-terminal d-c resistance measurement.

The d-c direct conductances of *G*_1_ and *G*_2_ are compared with the resistance of a stable 10^4^-ohm d-c resistor in the circuit of figure 17, which shows the provision for eliminating the effect of leakage resistances *R*_3_ and *R*_4_ to the shield. The bridge is balanced first with point (1) grounded, which shorts *R*_4_ and places *R*_3_ in parallel with *G*_1_; and then with point (2) grounded, which places *R*_4_ across the battery and places *R*_3_ across the 10^4^-ohm standard *R*#505. Neither balance depends upon *R*_4_, and a large *R*_3_ affects the two balances equally with opposite sign; hence the average balance gives the ratio^3^

$$\frac{r_{4}+1/G_{1}}{r_{3}+R\#505}=\frac{B}{{\bar{A}}_{1}}$$

Interchanging the link and standard, moving the galvanometer to *D*_1_ and repeating the two balances gives

$$\frac{r_{4}+R\#505}{r_{3}+1/G_{1}}=\frac{B}{{\bar{A}}_{2}},$$

so that to the accuracy desired $R\#505=1/G_{1}(\times \left[1+\left({\bar{A}}_{1}-{\bar{A}}_{2}\right)/2B\right]$, which allows the resistance of *G*_1_ to be compared with the resistance of the d-c standard. The measurement is made with *G*_1_ connected to the bridge center, and measurements are made between the same junctions used in the a-c measurement. A similar measurement of *G*_2_ allows the 10^4^-ohm d-c standard to be measured in terms of G¯=12(G1+G2), which is obtained from the a-c bridge. The measurement method involves the well-known double substitution technique with provision for eliminating the errors which might be caused by leakage resistance.

The variable arm *A* is constructed from a 100-ohm direct reading ratio set with a smallest step of one ppm, but is connected in series with a 900-ohm resistor to give a smallest step of one part in 10^7^. This 900-ohm resistor, a 10^3^-ohm resistor *B*, the 10^4^-ohm standard, and the link rest on a mercury stand in the oil bath.

Table 2 shows the results of several comparisons of the 10^4^-ohm d-c resistor with the 1-pf reference capacitor, using the frequency-dependent bridge. Recalling that the reference capacitor was measured in terms of the computable capacitor in May and July 1960, it will be noted that groups of runs were made immediately preceding and immediately following each cross capacitor measurement.

Each set of one cross capacitor measurement and two groups of resistance-capacitance bridge runs constitutes an evaluation of the resistance of the 10^4^-ohm standard *R*#505.

## 5. Frequency Dependence of Bridge Resistors

**Figure 18:**
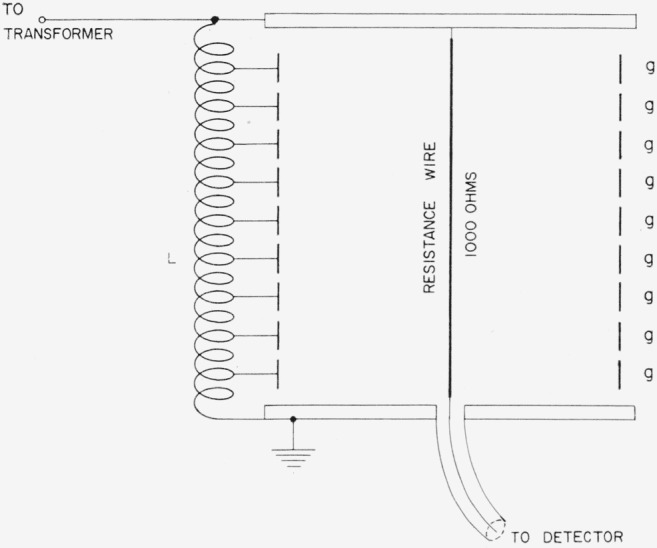
A-c–d-c resistance transfer standard.

The measurements of the a-c–d-c resistance differences of *G*_1_ and *G*_2_ were made by comparing them with a standard resistor of simple geometry having predictable frequency characteristics, using both direct current and 1592 c/s alternating current. Figure 18 shows the cross section of such a standard whose resistance element consists of a single straight wire 0.0008 in. in diameter and 9.5 in. long having a resistance of 1000 ohms. The structure is cylindrical with the resistance wire down the center. The large phase angle expected from a conventional coaxial line resistor is eliminated by the use of a set of guard rings, *g*, about 4 in. in diameter, which surround the resistance wire. The potentials of the guard rings are defined with a tapped inductor *L* placed between the line terminal and ground. The inductor taps are placed to provide a uniform potential gradient down the center of the cylinder in the absence of the resistance wire.^4^

When a bridge containing the transfer resistor is balanced with the resistance wire in place, the detector voltage is zero, and the potential gradient along the wire is still uniform. Since the wire does not change the electric field within the resistor assembly, we may expect no capacitance contribution to the resistor phase angle and a-c–d-c resistance difference. The effectiveness of the guard rings may be checked by connecting adjacent guards in parallel to cut the effective number of them in half, and by suitably reconnecting them to the tapped inductor. If the apparent resistance and phase angle measured in these two ways are the same, we may assume that the number of guard rings is sufficient to eliminate the effects of capacitance within the resistor. In practice it is found that even with all of the guard rings grounded, the measured a-c resistance is equal to the a-c resistance with all guards connected properly to the tapped inductor, and the phase angle change between the two-guard arrangements is less than 3 microradians.

The inductance of the standard resistor was measured by replacing the fine resistance wire with a larger low resistance wire and measuring, with a Maxwell-Wien bridge, the inductance including that of the coaxial leads between the junction points of the bridge center used in the measurements to be reported below. A simple computation [12] yields the difference between the inductances of the fine wire and the low resistance wire. We find for the series inductance of the resistor attached to the bridge center *L*=1.1 microhenries. This inductance has a negligible effect on the a-c resistance, but produces a phase angle of 11 microradians.

**Figure 19:**
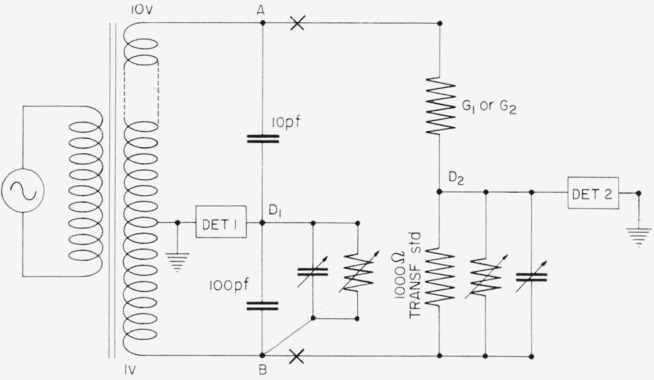
Circuit for comparing the transfer resistor with the bridge resistors, using ac.

Figure 19 shows the bridge used for the a-c comparison of the 1000-ohm transfer resistor, *R_T_*, with *G*_1_ or *G*_2_. The 10:1 transformer is the one used for the capacitance step-up, and has a known ratio. A balance is observed on detector 1 with the resistors disconnected at “X” to compare the 100-pf capacitor with the 10-pf capacitor. When the resistors are placed in the circuit and both bridges are balanced, a change in the balance at detector 1 indicates a change in the transformer ratio between the junctions *A* and *B*, and allows the loaded voltage ratio between these points to be calculated. This figure combined with the readings of the admittances required to balance detector 2 allows the a-c ratio of the resistances of *G*_1_ or *G*_2_ to the resistance of the 1000-ohm transfer standard to be determined, and also allows the phase angles of *G*_1_ and *G*_2_ to be determined from the known phase angle of the transfer standard.

**Figure 20:**
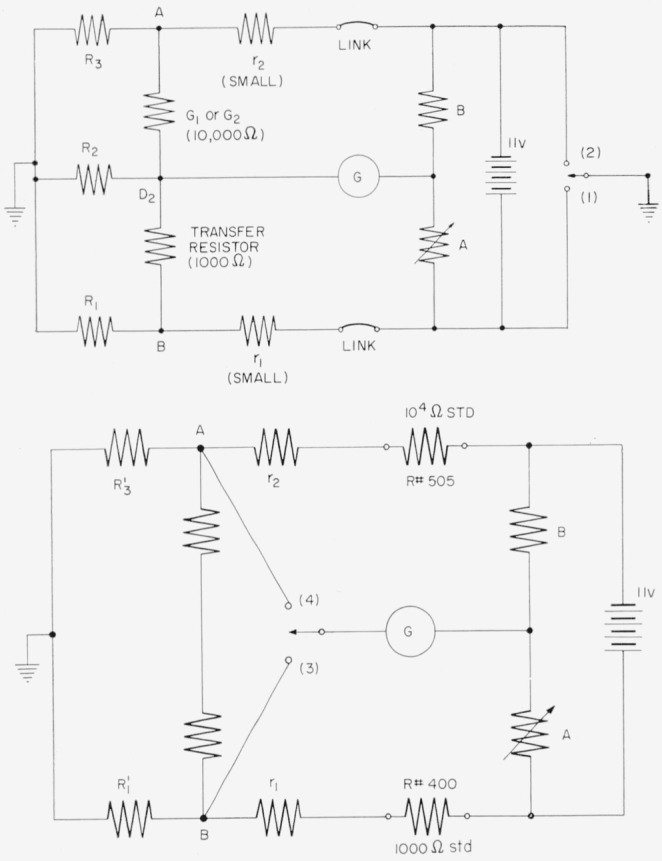
Circuits for comparing the transfer resistor with the bridge resistor, using dc.

Figure 20 shows the bridge used for comparing the d-c resistance of the transfer standard with *G*_1_ or *G*_2_. Since the tapped inductor of the transfer resistor presents a low d-c impedance to ground, it must be removed from the circuit for the d-c measurement. With this done, the leakage resistances *R*_1_,* R*_2_, and *R*_3_ are all high. Series resistances *r*_1_ and *r*_2_ in connecting wires are small. Two balances are made with points 1 and 2 grounded in turn. The direct reading ratio set readings are called *A*_1_ and *A*_2_, respectively.

The bridge is then connected as in figure 20, where *R*_1_*′* and *R*_3_*′* are low resistance “shorts.” The bridge is balanced with the galvanometer connected to 3 and 4 in turn; the readings of the DRRS are called *A*_3_ and *A*_4_ respectively. An analysis shows that for *G*_1_,

$$R_{T}G_{1}=\frac{R\#400}{R\#505}\left[1+\frac{100\left(A_{1}-A_{3}\right)}{11B}+\frac{10\left(A_{2}-A_{4}\right)}{11B}\right].$$

Provided that the resistance ratio of the d-c standards is known, measurements of this form yield the a-c–d-c differences of both *G*_1_ and *G*_2_. Since the 10^4^-ohm resistor #505 was used for both this measurement and for the direct d-c comparison between it and *G*_1_ and *G*_2_ in connection with the frequency dependent bridge, one finds that resistor #505 serves only to maintain the unit between the two measurements, and that in effect the 10:1 transformer is used five times to step up the impedance of the 1-pf standard capacitor to the 1000-ohm transfer resistor. The d-c step-up is then needed only from the bank of one-ohm reference resistors to the 10^3^-ohm d-c standard resistor #400. An error in the d-c step-up from 10^3^ ohms to 10^4^ ohms would cause no error in the final value assigned to the one-ohm reference resistors, but would only cause an error in the measured a-c–d-c differences of *G*_1_ and *G*_2_, which in this sense is not needed for our work. In fact, the only reason for including 10^4^-ohm #505 in the measurements is that it is much more stable than *G*_1_ *G*_2_, and the transfer resistor. The use of 10^4^-ohm #505 allows the complete determination to be broken up into smaller steps which may be completed more quickly and with higher accuracy. Subject to the above comments, we find that *G*_1_ and *G*_2_ have identical a-c–d-c differences and that their a-c resistances are 0.85 ppm higher than their d-c resistances. The average of their phase angles is found from the a-c measurement using the phase angle calculated for the transfer resistor to be 16.6 microradians (capacitive).

## 6. Conclusion

Combining the results of table 2 with the measured a-c–d-c difference of *G*_1_ and *G*_2_ we find *R#*505 = (10^4^ ohms−19.4 ppm). Comparison of this resistor with the NBS bank of one-ohm reference standards yields the value (10^4^ ohms−21.7 ppm) in terms of the ohm as adopted in 1948 [13] and as maintained at NBS. Our measurements therefore indicate that the NBS unit of resistance is 1.000002_3_ ohms.

Two values have been obtained for the phase angles of the 10^4^-ohm woven-wire resistors; 16.6 microradians from the computed phase angle of the straight wire a-c–d-c transfer resistor and 15.0 microradians based on the assumption that the 1-pf reference capacitor has no phase angle. The discrepancy of 1.6 microradians may be ascribed to the actual phase angle of the 1-pf capacitor, an error of 0.16 microhenry in the measurement of the transfer resistor series inductance, or a combination of these two effects. The agreement is considered satisfactory in view of the uncertainties in these measurements.

Table 3 summarizes the steps of this determination and lists the estimated uncertainties associated with each step. The estimates are in most cases little more than guesses, since they must include allowances for possible systematic errors. The estimates are in all cases given as “50 percent errors,” meaning that in the experimentor’s judgment the chances of the error being greater or less than this amount are approximately equal. It is judged to be almost certain that the error is less than four times the stated 50-percent error. No allowance is made for the possibility of error in the assigned value of the speed of light, which is taken as a predetermined constant, *c* = 2.997925×10^10^ cm/sec.

With the exception of the speed of light, all measurement uncertainties may be reduced with suitable refinements in measurement techniques. It is believed that with the development of an improved cross capacitor and with minor changes in the rest of the equipment, the measurement uncertainties not including the contribution from the speed of light may be reduced below 1 ppm.

## 7. Comparision With Other Determinations

**Figure 21:**
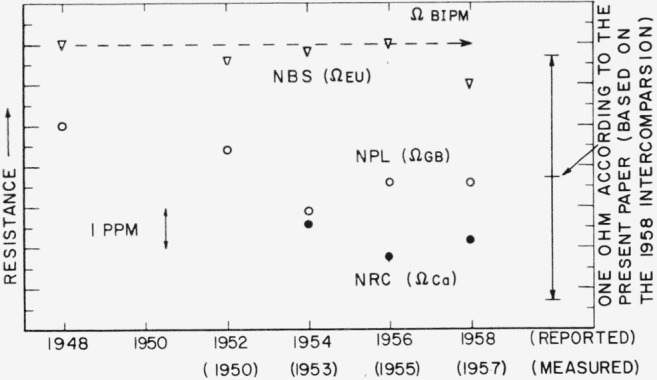
Resistance units maintained by NBS, NPL, and NRC in terms of the unit maintained at the International Bureau of Weights and Measures (Ω_BIPM_).

In order to compare several recent evaluations of the 1948 resistance unit with each other, it is necessary to know the relative values of the units in terms of which these evaluations were made. Figure 21 shows the results of comparisons made at the International Bureau of Weights and Measures (BIPM) of resistors embodying the units as maintained by NBS (Ω_EU_), NRC (Ω*_Ca_*), and NPL (Ω*_GB_*) in terms of the unit as maintained at the International Bureau (Ω_BIPM_) [14, 15, 16, 17].

No information is yet available concerning the 1960 intercomparisons, so it is impossible to compare the present work with Ω_BIPM_ at this time. For this reason and for consistency in the table to follow, all measurements will be referred to the unit as maintained at NBS, Ω_EU_.

Table 4 contains values assigned to Ω_EU_ by several recent investigations [18, 19, 20]. The values have been adjusted to allow for the difference between Ω_EU_ and the units used for the measurements. The results obtained by Thomas et al have been referred to Ω_EU_ instead of the international ohm, and also revised upwards by 3 ppm. This revision represents the results of a new evaluation of the current distribution in the primary winding of the mutual inductor, and is based on measurements of the dependence of resistivity upon strain in a sample of the wire used for the winding. These measurements were made in 1956 by Wells [21]. The revised current distribution correction was also calculated by him, but the result was not published.

## Figures and Tables

**Table 1:** Capacitance of cross capacitor

Based on 1-pf reference capacitor #NBS 89790-B.				

Time and date (1960)	Nominal length	Frequency	Measured capacitance	D-c capacitance

	*in.*	*c/s*	*pf*	*pf*
11:00 a.m. May 12	14	1592	1.3898231	1.3898241
8:55 a.m. May 13	14	1592	1.3898224	1.3898234
9:40 a.m. May 13	14	1592	1.3898222	1.3898231
2:05 p.m. May 13	14	1000	1.3898229	1.3898233
2:50 p.m. May 13	14	1592	1.3898220	1.3898229
1:20 p.m. May 16	4	1592	0.3973528	0.3973532
2:00 p.m. May 16	4	1592	.3973531	.3973535
2:55 p.m. May 16	4	1000	.3973536	.3973538
10:45 a.m. May 18	14	1592	1.3898201	1.3898210
11:35 a.m. May 18	14	1000	1.3898211	1.3898215
12:20 p.m. May 18	14	1592	1.3898198	1.3898208

Based on 1-pf reference capacitor #NBS 89790-B.				

Time and date (1960)	Nominal length	Frequency	Measured capacitance	D-c capacitance

	*in.*	*c/s*	*pf*	*pf*
9:22 a.m. July 21	14	1592	1.3898177	1.3898187
10:15 a.m. July 21	14	1000	1.3898177	1.3898180
12:45 p.m. July 21	14	1000	1.3898171	1.3898175
1:15 p.m. July 21	14	1592	1.3898164	1.3898174
9:45 a.m. July 25	4	1592	0.3973511	0.3973515
11:20 a.m. July 25	4	1000	.3973511	.3973513
1:00 p.m. July 25	4	1000	.3973512	.3973514
1:43 p.m. July 25	4	1592	.3973507	.3973511
9:15 a.m. July 27	14	1592	1.3898154	1.3898164
10:05 a.m. July 27	14	1000	1.3898165	1.3898168
12:52 p.m. July 27	14	1000	1.3898155	1.3898169
1:15 p.m. July 27	14	1592	1.3898165	1.3898175

Measured cross capacitance difference=0.9924687 pf.

Computed cross capacitance difference=0.9924151 pf.

True value of reference capacitor = 1-pf −54.0 ppm (May 1960).

Measured cross capacitance difference=0.9924661.

Computed cross capacitance difference=0.9924151.

True value of reference capacitor=1-pf −51.4 ppm (July 1960).

**Table 2:** Resistance of 10^4^ ohm d-c resistor #505

Based on 1 pf reference capacitor #NBS 89790-B neglecting a-c–d-c differences		

Date (1960)	*R*#505	Average phase angle of 10^4^ ohm a-c resistors

		*Microradians*
May 5	10^4^ ohms −73.6 ppm	+15.0
May 10	−73.4	+15.4
May 11	−74.3	+14.8
May 19	−74.1	+15.4
May 20	−74.0	+15.2

Average	−73.9 ppm	+15.2

Date (1960)	*R*#505	Average phase angle of 10^4^ ohm a-c resistors

		*Microradians*
July 18	10^4^ ohms −72.2 ppm	+14.7
July 19	−71.8	+15.0
July 28	−72.5	+14.8
July 29	−71.6	+14.7

Average	−72.0 ppm	+14.8

True value of 1 pf reference capacitor = l pf −54.0 ppm.

Resistance of *R*#505=10^4^ ohms −19.9 ppm.

True value of 1 pf reference capacitor = 1 pf −51.4 ppm.

Resistance of *R*#505=10^4^ ohms −20.6 ppm.

**Table 3:** Uncertainties associated with steps followed in this determination

	50% error

Measurement of the reference capacitor in terms of the computable capacitor, neglecting the contribution from the uncertainty in the speed of light	*ppm* 2.0
Instability of the reference capacitor	0.3
Ten-to-one step-up using alternating current (times 5)	.1
Frequency dependent bridge	.3
Dependence of bridge resistors upon frequency	.3
Step-down from 10^3^ ohms to 1 ohm using direct current	.3

Measurement uncertainty neglecting the uncertainty in the speed of light	2.1

**Table 4:** Values assigned to the resistance unit maintained at NBS (Ω_EU_) by several recent investigations

(See text.)			

Date of measurements	Reference	Laboratory	Value of the resistance unit Ω_EU_ maintained at NBS

1938 to 1949	Thomas, Peterson, Cooter, & Kotter	NBS	0.999997
1951	Rayner	NPL	1.000004
1957	Romanowski & Olson	NRC	.999996
1960	Present paper	NBS	1.000002_3_

## Footnotes

1 Figures in brackets indicate the literature references at the end of this paper.

2 Bridge networks of this type were investigated at the National Standards Laboratory in Sidney, Australia, in 1952 but the findings were not published. The network described in this paper was subsequently developed independently.

3 This relationship is actually an approximation, and depends upon R_3_ being large compared with *R*#505. Since the two values of A_1_ differed in our measurements by only a few parts per million, the approximation introduces no significant error.

4 Similar arrangements applied to high voltage transformer testing have been described by Silsbee [10] and Weiler [11].
